# An Argument for Medical Education of Dentists

**Published:** 1901-03

**Authors:** Eugene S. Talbot


### AN ARGUMENT FOR MEDICAL EDUCATION OF DENTISTS.

BY EUGENE S. TALBOT, M.D., D.D.S.

A TWENTY-SEVEN-YEAR-OLD woman came to me in November, 1900, through her physician, for advice and treatment. She is a proof-reader for a magazine. She weighs ninety-four pounds, and her assimilation is poor. She has cold hands and feet, and is awakened about three A.M. with cold extremities and cannot get to sleep except by wrapping her legs and feet in a woollen blanket. A year ago peritonitis from exposure to cold and coincident low vitality resulted, from which she recovered. Her mouth and teeth were so sensitive that eating was a torture. On application to a dentist, he, finding the teeth sensitive, applied arsenic and destroyed three pulps in small superficial cavities. The treatment did not seem logical to the woman, since every other cavity was as sensitive. The teeth in which the pulps had been removed were very sore. Upon tapping, the sound teeth were found equally painful with those whose pulps had been removed.

Interstitial gingivitis of a low type extended throughout the entire alveolar processes. The woman was placed upon tonics. The gums and alveolar process were treated with iodine. Her vitality is gradually being restored. The interstitial gingivitis is lessening and the teeth are recovering their normal condition.

Operations for filling, previously impossible, are now producing excellent results.

